# Unlocking Molecular Fingerprint of an Ombrotrophic Peat Bog: Advanced Characterization Through Hexamethyldisilazane Thermochemolysis and Principal Component Analysis

**DOI:** 10.3390/molecules29235537

**Published:** 2024-11-23

**Authors:** Sara Moghnie, Emil Obeid, Jalal Halwani, Laurent Grasset, Khaled Younes

**Affiliations:** 1 College of Engineering and Technology, American University of the Middle East, Egaila 54200, Kuwait; sarah.mughneyah@aum.edu.kw (S.M.); emil.obeid@aum.edu.kw (E.O.); 2 Water and Environment Sciences Laboratory, Lebanese University, Tripoli P.O. Box 6573/14, Lebanon; jhalwani@ul.edu.lb; 3 Université de Poitiers, Institut de Chimie des Milieux et Matériaux de Poitiers IC2MP, UMR CNRS 7285, Equipe Eaux, Biomarqueurs, Contaminants Organiques, Milieux, B27, 4 rue Michel Brunet, CEDEX 9, 86073 Poitiers, France

**Keywords:** thermochemolysis, hexamethyldisilazane, Principal Component Analysis, peatland

## Abstract

This study examines a boreal peatland (the Sagnes peatland, Fanay, Limousin, France) with a depth of 1 m. This peatland is currently in the late stages of organic deposition, as evidenced by the growth of *Carex* species, along with *Sphagnum* mosses, in the uppermost level. To gain molecular insights, we conducted an analysis of the lignin and polyphenolic counterparts using HMDS (hexamethyldisilazane) thermochemolysis, enabling the identification of lignin degradation proxies. The goal was to develop characteristic indicators for the state of lignin degradation based on the relative distribution of lignin phenols, measured by gas chromatography coupled with mass spectrometry (GC-MS) after the HMDS thermochemolysis. For that purpose, the singular contribution of the 11 aromatic moieties yielded, along with SGC (sum of lignin moieties) and the most lignin degradation proxies, were applied. It has been shown that HMDS thermochemolysis exhibited the capacity to reveal oxidized and degraded lignin fractions, following the increasing trend yielded for most moieties and SGC proxy, in the mesotelm and catotelm layers. In addition, the C/G (Cinnamyl/Guaiacyl) and S/G (Syringyl/Guaiacyl) ratios showed their highest input in the upper half of the core. This bias in the aforementioned ratios could indicate that HMDS thermochemolysis is to be applied for geological samples, where low G-compounds exist. For the sake of validating HMDS thermochemolysis’ application, Principal Component Analysis (PCA) was then applied to the molecular fingerprint. For ratios and proxies of aromatic moieties of HMDS thermochemolysis, the PCA approach exhibited a higher contribution (79%). This indicates the efficiency of these ratios in describing the molecular fingerprint of peat depth records. In addition, a higher separation between the contributions of the investigated variables (molecular proxies) along the first two PCs was noticed. In other words, the variables that showed a high contribution towards PC_1_ exhibited a low contribution towards PC_2_, and vice versa. These findings indicate the high reliance of applying the ratios and proxies of HMDS thermochemolysis.

## 1. Introduction

Soil organic matter (SOM) plays a vital role in the formation and dynamics of soil. Its composition is a characteristic of the soil’s origin. It comprises plant materials at different decomposition stages, as well as cells and tissues of soil microorganisms and their modified forms resulting from microbial degradation. From the geochemical perspective, most vegetable organisms are made up of the same fundamental chemical classes, including lignin/polyphenols, carbohydrates, and lipids [1,2].

Lignin is considered the second most abundant biopolymer found in nature and makes up 15–35% of the dry weight of vascular plants [1,3]. Lignin monomers serve as effective biomarkers for woody material. They provide a distinct fingerprint enabling vascular plants to distinguish between different plant source types, such as angiosperms and gymnosperms, and to determine their woody character [4]. Due to the complex nature of SOM, its characterization requires thermal and/or chemical analytical methods. The use of such techniques helps in understanding organic matter (OM) at the structural level as well as the processes involved in its formation and conversions in both soils and sediments [5,6].

Thermal methods are widely used in OM analysis to evaluate the thermal stability and decomposition patterns of various OM components [7,8]. Techniques such as thermogravimetric analysis (TG) and differential thermal analysis (DTA) allow for the study of OM by monitoring weight loss and thermal behavior under controlled temperature increases [7,8]. Studies have shown that these methods effectively differentiate components based on their thermal degradation profiles, especially in peat and lignite, where humic acids exhibit complex decomposition pathways influenced by the carbon and sulfur content [7,8].

Chemical molecular methods are fundamental in the analysis of OM as they provide insights into its composition, structure, and transformation processes. These methods include techniques such as alkaline oxidation, acid hydrolysis, and solvent extraction, each targeting specific biomolecular classes within OM [9]. Alkaline oxidation, often performed using CuO-NaOH, is widely used for lignin analysis as it breaks down complex lignin polymers into identifiable monomers, allowing for the differentiation of vegetation sources and lignin degradation states. Acid hydrolysis, primarily with hydrochloric acid, targets carbohydrates, breaking them down into simpler sugars to study their origins and potential microbial contributions [9]. Solvent extraction methods, such as the Bligh and Dyer method, isolate lipids, which are then analyzed to understand structural and energy-storing components within OM [9,10]. One major shortcoming of chemical methods in OM analysis is their specificity to particular components, which can limit a holistic understanding of OM’s complex structure [9,10].

To overcome the specificity limitations of traditional chemical methods in OM analysis, coupling them with thermal methods through thermochemolysis offers a robust solution. Thermochemolysis combines thermal decomposition with chemical derivatization, typically using alkylating, acylating, or silylating agents, to analyze various OM components simultaneously [10,11,12]. This approach enhances the detection of diverse molecular classes—such as lignin, carbohydrates, and lipids—by breaking down complex structures into smaller, methylated compounds that can be identified with high precision [10,11,12].

Thermally assisted hydrolysis and methylation (THM) is by far the most commonly used thermochemical approach [10,11,12]. It involves heating OM in the presence of a methylating agent like tetramethylammonium hydroxide (TMAH) to identify molecular components such as lignin, carbohydrates, and lipids. This technique, particularly useful for lignin analysis, selectively cleaves bonds within the lignin structure, allowing for the detailed analysis of lignin degradation and microbial contributions at varying peat depths. By applying such thermochemical techniques, researchers can gain insights into OM’s structural composition, enhancing our understanding of its ecological roles and degradation dynamics in soil and sediment environments.

THM is a chemolytic process that involves the cleavage of various bonds, including ether and ester bonds, as well as transesterification. Following this, methylation occurs, where COOH and OH phenolic groups are alkylated to form methoxyl groups. The agent involved in this process is TMAH reagent, which is considered to be a relatively strong base (pKb = 4.2), and it enables the analysis of both free and bound carboxylic and alcoholic moieties [6]. Thus, TMAH at high temperatures is found to be efficient in identifying monomers of biocomponents like cellulose, free/terminal sugar units of polysaccharides [6,13], lignin and tannins [14], as well as aliphatic lipids [15]. One advantage of THM is that it can considerably reduce the decarboxylation and dehydration that occur during pyrolysis, providing further information for the latter process [16]. Hence, THM provides a degree of protection for the oxygenated functional groups during the degradation process. Nevertheless, it remains unclear whether this process prevents the induction of secondary reactions. This could potentially give rise to the controversial presence of benzenecarboxylic acids that could pre-exist in the original sample, or alternatively, could result from disproportionations of aldehydes through Cannizaro reactions. These hypotheses were put forth following the pioneering work by Challinor [16].

Additionally, THM exhibits an inability to distinguish between bound and free carboxylic and alcoholic moieties that were initially present in samples or between ester bonds with varying reactivity [17]. Thus, this shortcoming was partially resolved using TMAH substitutes that require fewer reactive reagents [17]. For instance, the derivatization of free fatty acids using tetraethylammonium acetate (TEAAc) and its comparison with TMAH, which derivatized both bound and free fatty acids, revealed that the majority of the fatty acids in the humic acid samples were in their free forms, trapped within the organic network [18,19]. An alternative approach involves employing a silylating agent; HMDS has been suggested to be used for examining natural products resulting from polar molecules, such as proteins and lipids [20], carbohydrates [5,21], and humic acids [6]. Furthermore, bis (trimethylsilyl)trifluoroacetamide (BSTFA) has proven to be effective in the analysis of lignin [22]. Following our knowledge, no prior studies have showed, in a sophisticated way, lignin moiety dynamics along the peat core.

**Figure 1:**
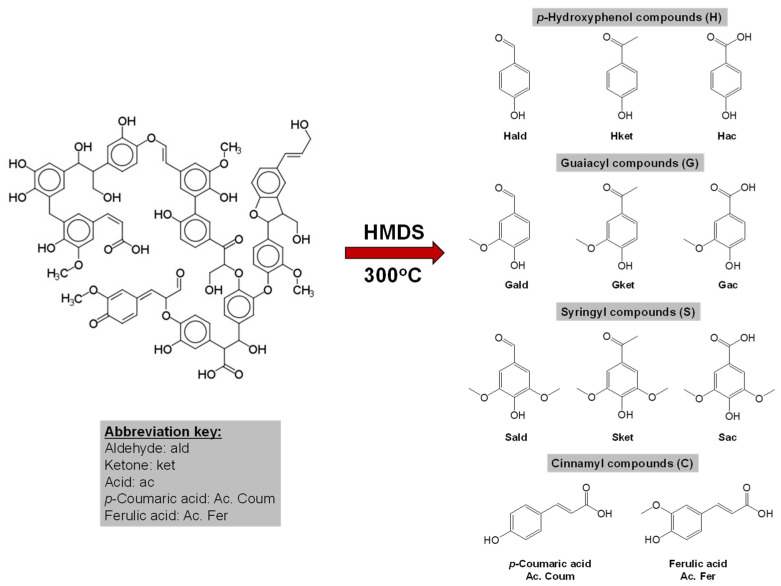
The origin of phenol monomers from lignin following HMDS thermochemolysis.

The aim of this study is to employ HMDS thermochemolysis coupled with GC–MS to decipher lignin moieties (Figure 1) in soils and sediments. To attain this purpose, the Principal Component Analysis (PCA) technique has been applied for the sake of statistically validating the yielded findings. The samples that we worked on were those of an ombrotrophic peatland. The reason for using peatland samples is the diplotelmic character of this ecosystem, meaning that it has two layers of soil with distinct characteristics. The upper half represents the “living” part, characterized by fresh organic matter input and microbial activity. In contrast, the bottom half constitutes the “dead” part, where decomposed OM is preserved, making peats a valuable reference for paleoenvironmental interpretations [9].

## 2. Results and Discussion

### 2.1. Elemental Analysis and HMDS Aromatic Moieties

Table 1 presents the trends in elemental analysis components as observed across the different six depth records. For carbon content (%C), the highest yield was found at the Acro-Upp, representing nearly 40% of the total dry mass of the peat sample. Three distinct patterns are evident: (a) the highest carbon output, at the top most depth sample; (b) a decreasing trend from the top vegetation layer to the base of the mesotelm (dropping from 26% to 21% between Acro-Bot and Meso, as shown in Table 1); (c) and a plateau at the core’s lower depths (%C stabilizing around 38% between Int_Meso-Cato and Cato). The high %C values in the bottom ecological layer suggest the effective preservation of OM in the anoxic core sections. For oxygen content (%O), a pattern similar to %C is observed, with the highest levels appearing at the top of the peat core, and a certain increase and stabilization at the bottom core. The nitrogen content (%N) shows a progressively decreasing trend, with two local peaks at the ecological layer interfaces (2% at both Int_Acro-Meso and Int_Meso-Cato; Table 1). These peaks, along with the highest %N at the core’s top, suggest notable microbial activity at these depths. In the case of hydrogen content (%H), the highest values appear within the catotelm (reaching up to 5% at Cato), indicating a buildup of aliphatic structures at these depths. The sulfur content (%S) displays a gradually decreasing trend, with one peak at the core’s bottom (4% at Int_Meso-Cato, Table 1), aligning with prior observations of sulfate-reducing microbial activity in this layer [9].

**Figure 2:**
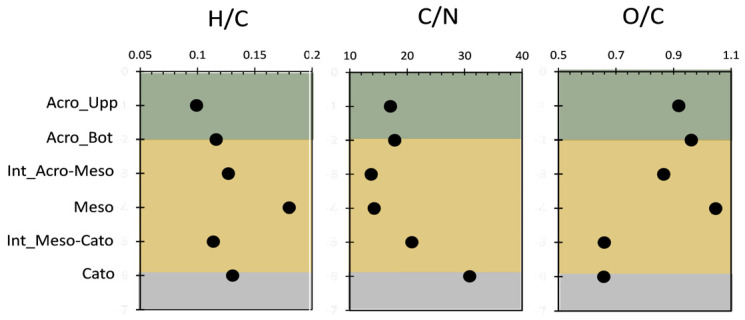
Elemental analysis ratios for investigated peat samples.

Figure 2 shows the elemental analysis ratios analyzed for the six investigated depth records. The H/C ratio serves as an indicator of aromaticity [9], and the lowest value was obtained in the acrotelm and catotelm. For the acrotelm, this is due to the input of polyphenolic materials from upper vegetations of the top of the peat core. For the catotelm, this is due to the preservation of recalcitrant lignin-rich OM in the anoxic layer [10]. In analyzing the C/N ratio within the peat core, the observed pattern of low C/N values in the acrotelm and mesotelm layers with a subsequent increase to high values in the catotelm provides important insights into the decomposition and OM characteristics at these different depths. Typically, low C/N ratios in the acrotelm and mesotelm suggest that these layers contain more decomposed OM, likely due to greater microbial activity facilitated by higher oxygen availability near the surface [9]. These conditions support a faster breakdown of OM, resulting in higher nitrogen relative to carbon [11]. Such environments align with nutrient-rich, oxygenated conditions where organic material decomposes more readily, reflecting a dynamic surface and middle layer that actively supports microbial decomposition [9]. Conversely, high C/N ratios in the deeper catotelm indicate a transition to less decomposed material rich in lignin and other resistant compounds [9,11]. This layer, being more waterlogged and anoxic, inhibits microbial activity, leading to a slower decomposition process and the preservation of plant material with a lower nitrogen content relative to carbon [9,11]. Such conditions favor carbon-rich, nitrogen-poor OM accumulation, making the catotelm an essential layer for long-term carbon storage in peat.

The observed pattern of high O/C values in the acrotelm and mesotelm followed by low values in the catotelm reflects the varying degrees of oxidation and decomposition across these layers. The high O/C ratios in the acrotelm and mesotelm suggest that these layers are more oxidized, likely due to increased exposure to oxygen near the surface [9,11,12]. Such conditions promote microbial activity, facilitating OM decomposition. These elevated O/C ratios typically indicate that organic material in these layers has undergone partial breakdown, which is consistent with aerobic conditions in the acrotelm and mesotelm [9]. In contrast, the low O/C ratios in the catotelm point to minimal oxidation, which is characteristic of the deeper, anoxic environment in this layer [9,11,12]. The restricted oxygen availability in the catotelm limits microbial breakdown, allowing for the better preservation of carbon-rich OM with lower oxygen incorporation. This low degree of oxidation results in lower O/C ratios and reflects the accumulation of lignin-rich, less-decomposed material.

**Figure 3:**
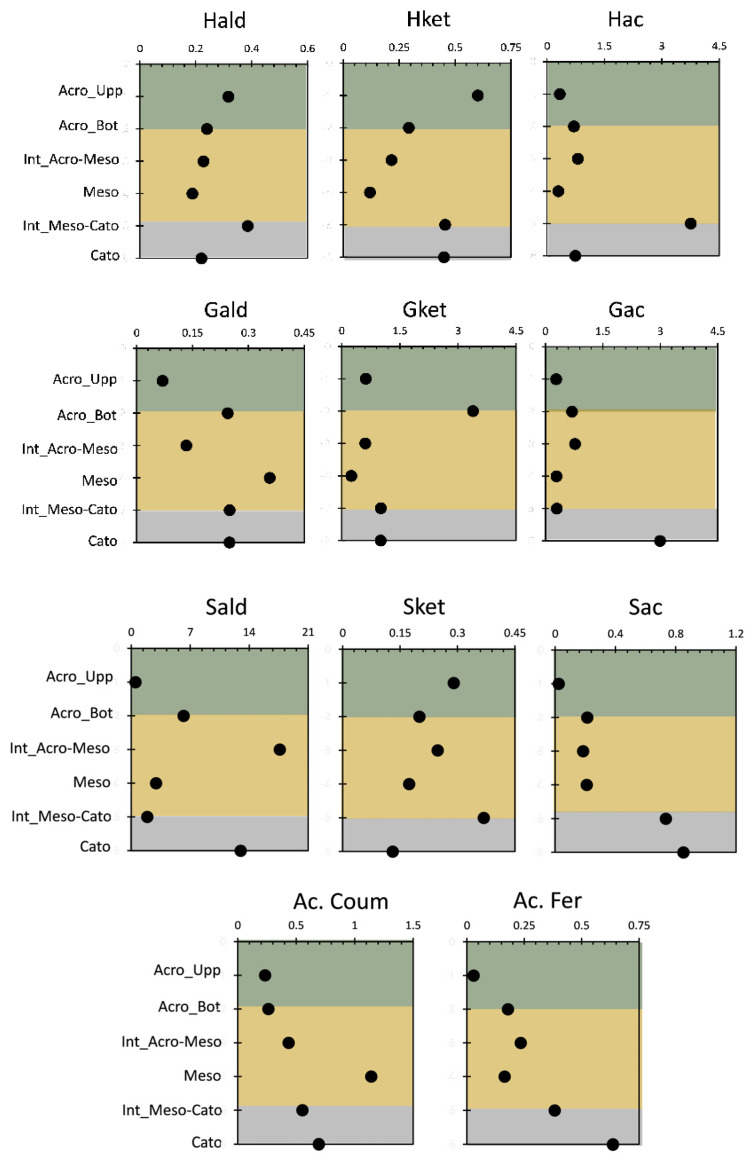
Depth profile (six peat core depth samples) of mass fractions for the 11 phenolic sub-units obtained by HMDS (mg·g^−1^ OC).

The HMDS thermochemolysis has yielded 11 aromatic moieties in the same way as the CuO-NaOH lignin oxidation procedure (Figure 1). This proximity between both methods indicates that the yielded phenols were more likely produced with the effect of thermal bond dissociation for both CuO-NaOH oxidation and HMDS thermochemolysis. The method yields a suite of one ring phenols (S, Syringyl; G, Guaiacyl; C, Cinnamyl; and H, *p*-hydroxyphenol) with their aldehyde, ketone, and acid counterparts. Figure 3 shows the depth profile of the different phenolic moieties, yielded by HMDS thermochemolysis. Unlike the case of TMAH thermochemolysis [10], the different moieties did not show nearly a decreasing profile along the peat core. This feature was only restricted to H aldehyde and ketonic moiety. These aromatic compounds showed a decrease in proportions (mg·g^−1^ OC (organic carbon)), interrupted by a stabilization in the bottom side of the core (Int_Meso-Cato and/or Cato; Figure 3).

For the rest of the yielded compounds, most of them showed the highest input in the bottom of the core, with the exception of Gald and Ac. Coum, that showed their highest input in the mesotelm. This could indicate a fungal activity that was yielded in this layer and showed its effect on the microbial reworking over deposited lignin [1]. These assertations could be confirmed by the prevalence of fungal activity biomarkers, when phospholipid analysis has been carried out on the investigated peat core [9]. In a previous study, it has been shown that possible actinobacterial activity could occur in the mesotelm following the input methylated fatty acids, yielded by the phospholipid analysis (PLFA) [9]. For Gket and Sald, the highest input has been shown at Acro_Bot and Int_Acro-Meso, which could probably indicate the occurrence of the microbial reworking activity of the fresh OM yielded from the uppermost vegetation. Hence, the highest PLFA input was scored in the highest part of the core (acrotelm and its interface with the mesotelm [9]). In brief, yielded aromatic moieties have shown multiple trends along the depth of the peat samples, which marks less consistency if compared with the TMAH thermochemolysis [10]. Interestingly, most of the moieties did not show their highest value at the uppermost peat sample, indicating that HMDS thermochemolysis is more suited to reveal oxidized and more ancient lignin forms. In order to decipher more findings with regard to these moiety dynamics, an overlook of the lignin conventional ratios would be more efficient.

### 2.2. Vegetation and Degradation Proxies

A number of distinctive markers for the lignin degradation profile were developed by analyzing the relative distribution of lignin phenols measured chromatographically following the HMDS analysis. Lignin proxies were used as described by Filley et al. [14], with all phenols normalized to 1 g OC. In general, the SGC content reduces as soil and sediments lignin degradation increases [23].

In our case, the SGC content showed an increasing trend from the upper part of the core to the interface between mesotelm and acrotelm. This could reflect the low degraded lignin in the upper part. The lowest SGC for the upper part has been noticed for the uppermost vegetation sample; this could indicate high lignin reworking due to the abundance of the microbial activity at the top following the accessibility of O_2_. The highest amount of PLFA moieties in the uppermost part of the core and obtained from a previous study [9] confirms the yielded findings in our case. These findings are in contradiction with the TMAH thermochemolytic profile, which highlighted the highest output of the upper half of the column at the uppermost vegetation record [10]. This discrepancy confirms that HMDS is not capable of revealing the fresh ligneous OM input as the TMAH and reflects the capacity of HMDS to reveal microbially degraded lignin in a clearer way. For the mesotelm, a low input of SGC content was noticed; this is due to the higher degradation rate of the lignin content as well as microbial oxidation and the access of oxygen in the mesotelm region, while this layer is in the emerged situation [10] since SGC yields might vary depending on the degree of lignin structure alteration and for different plant species. It is crucial to use it along other ratios in order to remove any bias that might result from the decline in the SGC profile [1].

**Figure 4:**
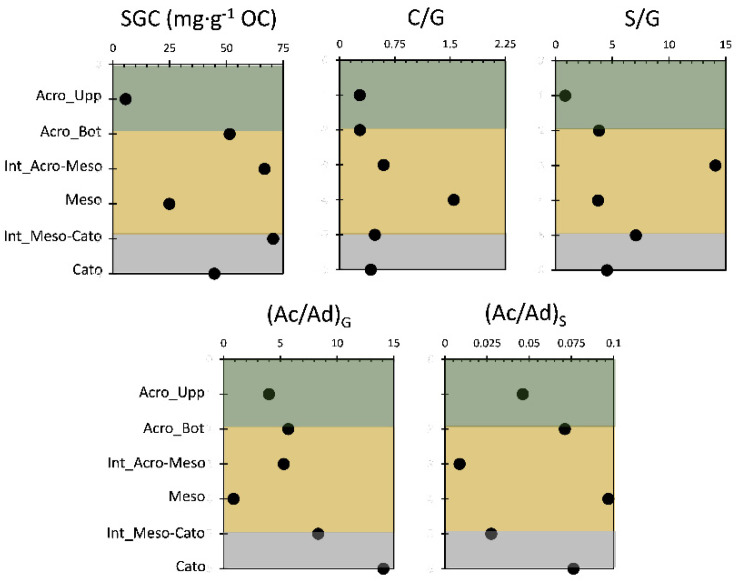
Depth profile (six peat core depth samples) for different ratios of the phenolic sub-units yielded by HMDS.

The S/G and C/G ratios indicate lignin type, as gymnosperms and angiosperms exhibit different distributions of G- and S-type lignin [1]. Gymnosperms primarily have G-type lignin, while angiosperms contain both G- and S-type units in similar proportions [1,23]. The C/G ratio suggests a higher contribution of non-woody plant material [24]. During lignin degradation, S- and C-units degrade faster than G-units, leading to reduced S/G and C/G ratios, except in the initial degradation stage [25]. Thus, these ratios are not often used as indicators of lignin degradation due to their opposing trends and overlapping source variations [25,26]. Some studies, however, have used S/G ratios to track lignin degradation in comparable vegetation types [1,10,27]. In this study, the highest S/G and C/G ratios were found in the mesotelm (Figure 4), likely due to significant leaf and root input during this stage of peatland formation or the selective decay of S moieties with the potential demethylation of methoxy groups on S-type lignin phenols [1,10].

Our study showed an increasing profile in both S/G and C/G ratios in the upper half of the core, particularly in the mesotelm region. The S/G ratio peaked at the acrotelm–mesotelm interface, indicating woody vegetation at that depth, consistent with the rise in the SGC content (Figure 4). In contrast, the increase in the C/G ratio from the acrotelm–mesotelm interface to the mesotelm suggested non-woody vegetation in the lower core [10]. Two explanations were proposed: the insufficient extraction of G-compounds by HMDS or a higher degradation rate of G-compounds during HMDS thermochemolysis [10]. The first was dismissed due to distinct moiety trends shown in Figure 3, indicating that HMDS should be used when the G-compounds are minimal.

The Ac/Ad ratios present a more efficient indicator of lignin degradation [1]. Studies have shown that the acid/aldehyde ratios (Ac/Ad) within three lignin phenol groups can be used as an indicator for identifying diagenetic changes in various geochemical samples. Thus, it is crucial to consider lignin’s diagenesis before analyzing its origin [10]. A high (Ac/Ad)G ratio was noticed in the lowest part of the peat core, especially in the mesotelm–catotelm interface to reach its peak in the catotelm. This indicates the capacity of HMDS thermochemolysis to reveal an ancient fraction of degraded lignin. The high values obtained in the interface reflect an increase in microbial oxidation, which is reflecting earlier lignin oxidation during peatland accumulation. The (Ac/Ad)S increased from the upper acrotelm to the bottom of the acrotelm to give the highest input then in the mesotelm. This might be due to an increase in microbial oxidation with depth due to the access of oxygen while the mesotelm layer emerged. Furthermore, it showed a high level in the catotelm; these results are consistent with the results obtained with TMAH following lignin degradation [1,28,29].

### 2.3. Statistical Analysis

The application of PCA in geochemistry spans across three distinct categories, showcasing its versatility in elucidating complex relationships within environmental systems. Firstly, PCA has been instrumental in determining the sources of organic matter (OM) by leveraging biomarker specificity, distinguishing between terrigenous and marine origins. This approach provides a valuable tool for tracing the fate of OM within terrestrial and aquatic environments [30]. Secondly, it aids in revealing similarities and dissimilarities among samples, offering a comprehensive understanding of compositional variations [10]. Thirdly, the application of PCA extends to uncovering intricate relationships among variables through the development of data-driven proxies, such as “degradation indices” [11,12,31,32].

Specific studies have delved into the multidimensional statistical analysis of molecular fingerprints in peat. Schellekens et al. [12,33,34] utilized the factor analysis method to extract parameters crucial for interpreting vegetation changes, anaerobic and aerobic decomposition, as well as fire incidence in various peat bogs. In a unique context, our study applies PCA to compare ombrotrophic peat samples and evaluate several depth records along the peat core. By leveraging this technique, we aim to unravel nuanced patterns and trends that may provide insights into the comparative effectiveness of HMDS thermochemolysis as a molecular degradation method for molecular analysis. This approach contributes to the broader understanding of environmental processes and aids in refining strategies for peat samples’ characterization [10]. 

#### 2.3.1. PCA for Relative Contribution of Aromatic Moieties

**Figure 5:**
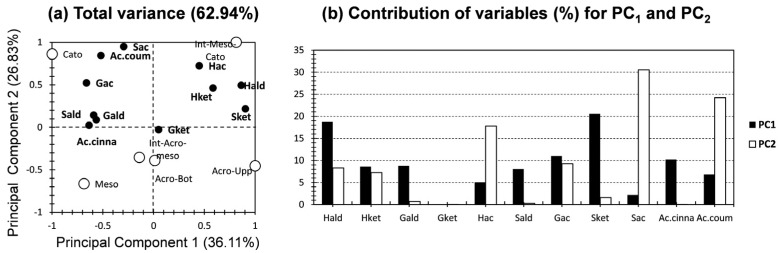
(**a**) PC_1_ vs. PC_2_ representation of datasets for the 11 aromatic moieties yielded by HMDS thermochemolysis (white bullets (individuals) present the six peat core depth samples, and black bullets (variables) present the yielded aromatic compounds). (**b**) % contribution of the investigated variables relative to PC_1_ (black) and PC_2_ (white).

Figure 5 shows the PCA for the yielded aromatic moieties by HMDS thermochemolysis. The first two PCs exhibited 63% of the total variance (36% and 27% for PC_1_ and PC_2_, respectively; Figure 5a). For the variables, the investigated moieties showed more alike equally distributed proportions, spanning from moderate to low contributions, along PC_1_ (Figure 5b). The highest contributions were yielded for Sket and Hald (scoring 21% and 19%; Figure 5b). These trends indicate the efficiency of the investigated moieties to reveal OM dynamics along a peatland as they share near amounts of contributions towards the dataset in-hand [35]. An exception arises for Gket, as it exhibited negligible contributions towards both PCs (Figure 5b), and was plotted near the node in Figure 5a. For PC_2_, the highest contribution has been scored for Sac and Ac. Fer (scoring 31% and 24%, respectively; Figure 5b). A moderate contribution was yielded for Hac (18%; Figure 5b). For the rest of the variables, minor to negligible contributions were scored.

For the individuals (peat core depth records), a high segregation can be noticed along the bi-plot, indicating a high degree of dissimilarity between them. These findings are expected, giving that the samples present different ecological features (input/preservation of OM, oxic/anoxic conditions, water level following seasons and depth) [9,36]. Interestingly, high proximity can be noticed between Acro-Bot and Int_Acro-Meso. For the Acro_Upp sample, it presented high positive and moderate negative contributions along PC_1_ and PC_2_, respectively (Figure 5a). This sample peculiarly showed a high influence by Sket and Hald along PC_1_, with no particular influence for any of the variables along PC_2_. For the Acro-Bot and Int_Acro-Meso sample, they exhibited a moderate negative influence along PC_2_, with no influence along PC_1_. The score of these samples is influenced by the highest loading factor for Gket. No specific findings could be deducted for this pattern, as Gket exhibited low contributions in our case. For the mesotelm sample, it showed a high negative influence along both PCs, and the score of this sample is influenced by the highest loading factor of Sald, Gald, and Ac. Coum along PC_2_ (Figure 5a). Regarding the PCA, the bottom core samples showed peculiar patterns, as the Int_Meso-Cato and Cato samples were oppositely plotted from one to the other. The first showed a high positive influence along both PCs and yielded a positive contribution with H-compounds. For the second, it showed a high positive and negative influence along PC_2_ and PC_1_, respectively (Figure 5a). The Cato sample showed a moderate positive correlation along Sac, Ac. Fer, and Gac. This indicates a higher rate of oxidation for the first peat deposition period [36]. These findings could be ascertained by the high contribution of both Ac/Ad ratios in the deepest peat sample (Figure 4).

In brief, the application of PCA for the aromatic moieties of HMDS thermochemolysis yielded an acceptable contribution, indicating the efficiency of this thermochemolytic technique for describing the molecular fingerprint of peat depth records. Interestingly, PC_1_ showed a more alike, even distribution contribution for the investigated variables; this is unlike PC_2_, where its influence was more likely skewed by Sac and Ac. Fer, as well as Hac, but to a lower extent. This indicates that even though it is known that PC_1_ exhibits the highest variance, PC_2_ was more efficient in discerning disparities between the investigated samples. The low clustering yielded, in the case of depth samples (individuals), indicated the high dissimilarity between them and ascertained the efficiency of yielding a comparison between the proposed depth samples.

#### 2.3.2. PCA for Proxies of Aromatic Moieties

**Figure 6:**
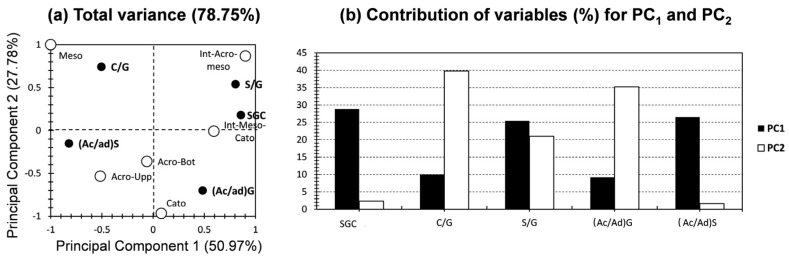
(**a**) PC_1_ vs. PC_2_ representation of datasets for the 5 aromatic proxies yielded by HMDS thermochemolysis (white bullets (individuals) present the six peat core depth samples, and black bullets (variables) present the proxies). (**b**) % contribution of the investigated variables relative to PC_1_ (black) and PC_2_ (white).

Figure 6 shows the PCA for the yielded proxies of aromatic compounds by HMDS thermochemolysis. The first two PCs yielded a higher variance than the relative contribution of moieties (Figure 6a), scoring 79% (51% and 28% for PC_1_ and PC_2_, respectively; Figure 6a). This indicates the higher efficiency of the presented ratios and proxies to decipher molecular fingerprint findings compared to the conventional way of seeking the relative contribution of each one of the yielded biomarkers. For the variables, the investigated ratios showed a higher discrepancy of contributions along the first two PCs. In fact, SGC and (Ac/Ad)S showed the highest contributions along PC_1_, scoring 29% and 27%, respectively (Figure 6b). The rest of the ratios exhibited low contributions, with the exception of S/G, for which there was a moderate contribution along both PCs. For PC_2_, the highest contributions were yielded for C/G and (Ac/Ad)G, scoring 40% and 35% (Figure 6b). The rest of the variables, except for S/G, yielded negligible contributions, with respect to PC_2_. This high discerning between contributions of variables, along with the higher total variance for the PCA of proxies, ensured a higher integrity for ratios if compared with the relative contribution’s approach. 

As the individuals assured similarly to the relative contribution’s approach (Figure 6a), a high segregation can be noticed for the ratios and SGC proxy along the bi-plot of the first two PCs (Figure 6a). This indicates a high degree of dissimilarity between the investigated samples and hence further ascertains the efficiency of the adopted sampling technique. These findings are in high accordance with the geological and ecological features of a peatland formation and deposition [9,36]. This high proximity has been yielded, in this case, for Acro-Upp and Acro-Bot samples. The latter presented the highest proximity with (Ac/Ad)S and showed a moderate negative influence, with respect to PC_2_, and no influence or a moderate negative influence towards PC_1_ (Figure 6a). For Int_Acro-Meso, this showed a high positive correlation along both PCs, and the score of this sample is influenced by the highest loading factor of S/G. In the PCA, SGC was more able to be influencing, mostly in terms of the SGC proxy that showed a positive correlation along PC_1_, with no correlation along PC_2_. The mesotelm sample was plotted on the positive and negative sides of PC_2_ and PC_1_, respectively, and showed a moderate influence by the C/G ratio. Similarly, to the case of PCA for relative contributions of moieties, a high dispatchment was noticed for the bottom core samples, as Int_Meso-Catotelm was exclusively and positively influenced by PC_1_, and the catotelm sample had its exclusive influence by the negative side of PC_2_ (Figure 6a).

In brief, the application of PCA for the ratios and proxies of the aromatic moieties of HMDS thermochemolysis yielded a higher contribution, indicating the efficiency of the thermochemolytic technique, and the data curation as ratios and indexes, for describing the molecular fingerprint of the peat depth records. This efficiency has been highlighted by a moderate to high total variance of both PCA approaches (Figure 5 and Figure 6) in comparison with the previous geochemical investigations using PCA for peat sample analysis [9,12,33]. In addition, a higher separation between the contributions of the investigated variables, along the first two PCs, was noticed. These findings indicate a higher reliance of applying the ratios and proxies of HMDS thermochemolysis.

## 3. Materials and Methods

### 3.1. Peatland and Geological Settings

The Sagnes peat bog is situated in the Limousin region of central France, where the subsurface is predominantly composed of igneous rocks, particularly granite. This peat bog is qualified topogenous, being supplied with water from a river, “Ruisseau des Sagnes”, which flows through the upper layer of soil. Granite extends beneath both the analyzed peat deposit and its surrounding area. The investigated peat is classified as a bog type (ombrotrophic) because its accumulation persisted above the surface level of the surrounding soil [9,37].

In the early stages of its development, vascular plants, including sedges and reeds, grew along the periphery of a topographic depression. As these plants reached the end of their life cycle, their debris have been buried at the depression’s bottom. The accumulation of vegetation gradually filled the entire topographic depression, leading to its transformation into a fen. Subsequently, *Sphagnum* mosses settle on the fen’s surface. Over time, as these mosses died and accumulated, they played a pivotal role in transforming the fen into a bog [9,38]. Along with the remaining moss vegetation, at the uppermost level of the peat core, vascular vegetation (mainly *Carex*) started growing, indicating that the Sagnes peatland is in its latest phases of development.

From a hydrological perspective, peatlands are sub-divided into different depth layers based on their water content. A peat bog is commonly referred to as “diplotelmic”, indicating that it consists of two layers of soil with distinct characteristics [36]. The peat located above the shallowest water table, maintaining unsaturation throughout the year, is referred to as the acrotelm. This layer constitutes the upper active zone containing roots and recently decomposed plant material. In contrast, the peat positioned below the deepest water table, remaining permanently saturated, is known as the catotelm. This layer represents the most decomposed anoxic portion. The intermediate region between these two layers is called the mesotelm, representing the layer where water table fluctuations occur. Hence, it will be emerged during dry seasons and submerged during wet seasons, making it a characteristic layer for microbial oxic/anoxic biota [36].

### 3.2. Peat Core and Sampling Strategy

**Figure 7:**
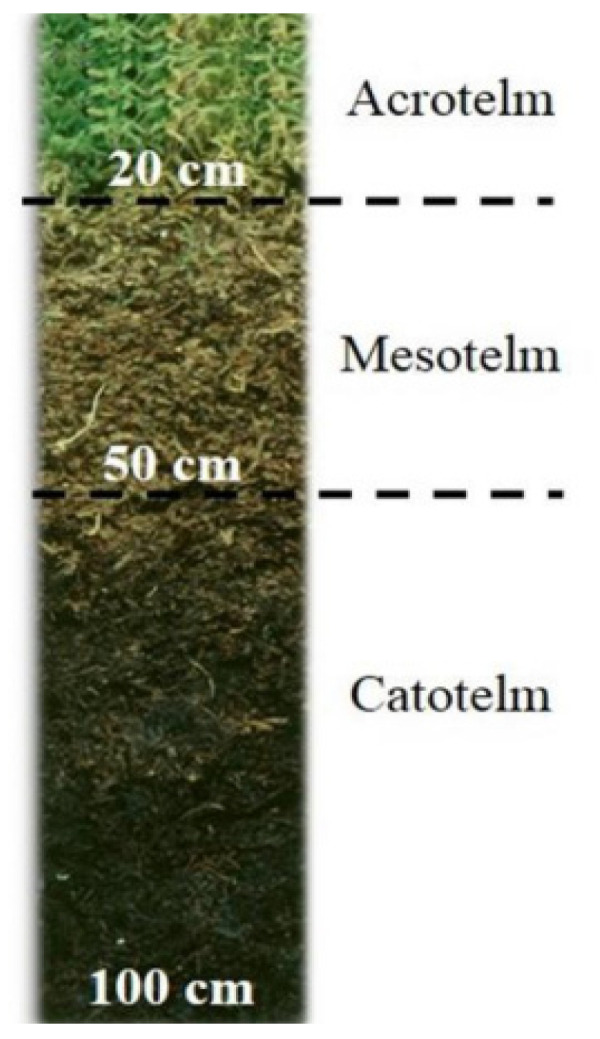
Peat core and depths of the three ecological layers.

Samples from three peat cores, each measuring 1 m in length, were collected in November 2012. These cores are the vertices of an equilateral triangle with sides of two meters. Subsequently, the cores were segmented into 4 cm slices and subjected to freeze-drying. Sampling involved collecting five samples from the upper oxic part (acrotelm; 0 to −20 cm), eight samples from the water table zone (mesotelm; −20 to −50 cm), and eleven samples from the anoxic part (catotelm; −50 to −100 cm) (Figure 7). In a previous study, we showed a more sophisticated description of sampling and condition methods [16]. The samples of this study were combined into six depth samples: (1) Acro-Upp, the upper part of the acrotelm; (2) Acro-Bot, the bottom part of the acrotelm; (3) Int_Acro-Mesto, the interface between acrotelm and mesotelm; (4) Meso, the mesotelm peat sample; (5) Int_Meso-Cato, the interface between mesotelm and catotelm; and (6) Cato, the catotelm peat sample.

Based on the von Post scale, the peat samples display a gradient of decomposition that reflects their position within the peat profile. In the acrotelm, the peat contains discernible plant leaves from the uppermost vegetation, indicating a low decomposition level. This layer likely corresponds to H^2^ to H^3^ on the von Post scale, where the structure of plant material is largely intact, and the peat exhibits minimal decomposition. As we move to the bottom of the acrotelm, the peat is light brown, suggesting slightly more decomposition yet still retaining recognizable plant fibers, likely falling into the H^3^ to H^4^ range, where the peat remains moderately fibrous and the structure is partially maintained. In the mesotelm, the peat samples are brown, showing a further progression in terms of decomposition. This layer corresponds to H^4^ to H^6^ on the von Post scale, indicating moderate decomposition. At this stage, plant structures become less distinguishable, and there is a greater presence of humic material with some darkening, but the peat still retains a mix of recognizable and amorphous components. Finally, the catotelm samples are dark brown, reflecting significant decomposition. These samples likely fall into the H^7^ to H^9^ range on the von Post scale, characterized by heavy decomposition, with the prevalence of some fibrolytic material. The peat is predominantly amorphous, suggesting minimal oxygen exposure and a high preservation of carbon-rich organic material, typical of the deep, anoxic layers conducive to long-term carbon storage.

### 3.3. Elemental Analysis

Carbon (C), hydrogen (H), and nitrogen (N) concentrations were analyzed through flash combustion at 960 °C, while oxygen (O) concentrations were determined through oxidation at a high temperature (1000 °C). The gasses produced were separated using gas chromatography (GC) and analyzed with total conductivity detection (TCD). All the analyses were performed with an elemental analyzer (Flash EA 1112; Thermo Scientific Instruments, Waltham, MA, USA). In this, the C content was used to normalize the yielded aromatic moieties and to correct the contribution from the mineral matrix.

### 3.4. HMDS Thermochemolysis

The thermochemolysis procedure follows the off-line method developed by Grasset and Amblès [39]. In this process, 50 mg of the samples was milled and mixed with 4 mL of HMDS. The furnace was heated to 300 °C for a duration of 30 min, with helium serving as the inert driving gas. The resulting pyrolysates were condensed in a cold trichloromethane receptacle. HMDS is used as a silylating agent for the thermally assisted derivatization of pyrolysis products (pKb = 6.45 weak base) due to its ability to silylate compounds. Consequently, all labile hydrogens, including those in alcohols and carboxylic acids, are silylated, simplifying the chromatographic characterization process. The various molecules resulting from this process were characterized using GC/MS equipment from Thermo Fisher Scientific (Waltham, MA, USA).

### 3.5. GC-MS

The GC-MS analysis was conducted for the identification of different compounds using a Trace GC instrument (Thermo Finnigan, Thermo Scientific) coupled to a mass spectrometer (Thermo Finnigan Automass). The injector (Thermo Finnigan PTV) operated at 250 °C, and a fused silica column (Supelco Equity 5%, 30 m × 0.25 mm i.d., 0.25 µm film thickness) with helium as the carrier gas (1 mL min^−1^) was employed. The oven temperature was programmed from 60 to 300 °C (held for 15 min) at a rate of 5 °C min^−1^. The MS instrument operated in the electron ionization mode at 70 eV, and ion separation utilized a quadripolar mass filter. Each analysis employed the full scan mode (*m*/*z* 50–650, 2 scans·s^−1^). Compound identification was based on GC retention times, mass spectra (compared with standards), and relevant literature data (see Younes et al. [9]).

### 3.6. PCA 

PCA aims to decrease the complexity of the problem by converting a large set of input variables into smaller features called principal components (PCs). However, the reduction in the number of variables may impact accuracy, creating a trade-off between accuracy and problem simplicity [35,40]. Nonetheless, the primary goal of PCA is to simplify the problem by diminishing the number of variables while retaining most of the information from the input features to maintain optimal accuracy. PCA accomplishes this by generating new uncorrelated variables (PCs) through a statistical process that performs an orthogonal transformation of the potentially correlated input features [35].

PCs are defined as directions that maximize the variance of the projected observation data along the principal subspace, minimizing information loss without significantly impacting accuracy. It can be demonstrated that PCs are arranged in a specific order based on the variance of the projected data. The goal of PCA is to maximize information in the first PC, equivalent to the direction that maximizes the variance of the projected data. Subsequently, the next maximum remaining variance is set in the second PC, and so on [35]. In this study, the PCA was conducted using XLSTAT 2014.5.03 software (Addinsoft, Paris, France) following the approach outlined by previous studies [40,41]:

(1) $$Fi=U_{j}^{T}M=\sum_{i=0}U_{ji}M_{i}$$

where *U* is the loading coefficient and *M* is the data vector of size n. The variance matrix *M*(Var(*M*)) is obtained by projecting *M* to *U* and should be maximized following:

(2) $$Var\left(M\right)=\frac{1}{n}\left(UM\right){\left(UM\right)}^{T}=\frac{1}{n}{UMM}^{T}U$$

(3) $$MaxVar\left(M\right)=Max\left(\left(\frac{1}{n}\right)\;{UMM}^{T}U\right)$$

Since $\frac{1}{n}{MM}^{T}$ is the same as the covariance matrix of *M*(cov(*M*)), Var(*M*) can be expressed by the following:

(4) $$Var\;\left(M\right)=U^{T}cov\;\left(M\right)\;U$$

The Lagrangian function can be defined by performing the Lagrange multiplier method by the following:

(5) $$L=U^{T}$$

(6) $$L=U^{T}cov\left(M\right)U-\delta (U^{T}U-I)$$

For (7), “*U*^T^*U*-I” is considered to be equal to zero since the weighting vector is a unit vector. Hence, the maximum value of var(*M*) can be calculated by equating the derivative of the Lagrangian function (*L*), in respect to *U*, by the following:

(7) $$\frac{dL}{dU}=0$$

(8) $$cov\left(M\right)U-\delta U=\left(cov\left(M\right)-\delta I\right)U=0$$

where δ is the eigenvalue of *cov*(*M*) and *U* is the eigenvector of *cov*(*M*)

## 4. Conclusions

In this study, we have applied HMDS thermochemolysis for the identification of aromatic molecular moieties in peat samples. The SGC content showed an increasing trend from the upper part of the core to the interface between mesotelm and acrotelm. This confirms that this technique is not capable of revealing the fresh ligneous OM input and reflects its capacity to reveal microbially degraded lignin in a clearer way.

Both the S/G and C/G ratios show an increasing profile in the upper part of the core, particularly within the mesotelm region; notably, the S/G ratio rises from the upper part of the acrotelm, reaching its highest input at the acrotelm–mesotelm interface. Two possible explanations could account for these trends: either the G-compounds are insufficiently “peeled” by the HMDS thermochemolysis process and its associated experimental conditions or the G-compounds experience a higher degradation rate compared to the S- and C-compounds during HMDS thermochemolysis. The first hypothesis is likely negligible, given that HMDS thermochemolysis produced distinct trends for each moiety, suggesting that HMDS may be the most applicable when G-compounds are low in the target sample. However, the data do not allow for a definitive conclusion on which mechanism—or a combination of both—might explain the observed S/G ratio values as this ratio is influenced by various factors, such as hydromorphism, fire events, original material composition, microbial activity, and abiotic reactions. These variables are complex and challenging to control, making the interpretation of S/G ratios inherently nuanced.

For lignin degradation proxies, a high (Ac/Ad)G ratio was noticed in the lowest part of the peat core, especially in the mesotelm–catotelm interface to reach its peak in the catotelm. This indicates the capacity of HMDS thermochemolysis to reveal an ancient fraction of degraded lignin. The high values obtained in the interface reflect an increase in microbial oxidation, which is reflecting earlier lignin oxidation during peatland accumulation. (Ac/Ad)_S_ showed an increasing trend, along the acrotelm, and showed the highest input in the mesotelm. This might be due to an increase in microbial oxidation with depth due to the access of oxygen while the mesotelm layer emerged.

In order to reach more sophisticated findings of HMDS thermochemolysis, the aromatic molecular fingerprint has been subjected to PCA. Two PCAs were made, for the individual inputs of 11 aromatic moieties, and for the different ratios employed. For the aromatic moieties, PCA yielded an acceptable contribution (63%), indicating the efficiency of this thermochemolytic technique for describing the molecular fingerprint of the peat depth records. The low clustering yielded for the depth samples indicates the high dissimilarity between them and ascertains the efficiency of making a comparison between the proposed depth samples. For ratios and proxies of aromatic moieties of HMDS thermochemolysis, PCA yielded a higher contribution (79%), indicating the efficiency of these ratios and indexes in describing the molecular fingerprint of the peat depth records. In addition, a higher separation between the contributions of the investigated samples, along the first two PCs, was noticed. These findings indicate a higher reliance of applying the ratios and proxies of HMDS thermochemolysis.

## Figures and Tables

**Table 1:** Details of investigated depth profile (six peat core depth samples) and elemental composition.

Samples Designation	Description	Depth (cm)	N	C	H	O
Acro-Upp	Upper vegetation with underlying peat samples	4	2.36	40.27	3.99	36.94
Acro-Bot	Acrotelm samples	12	1.45	25.80	3.00	24.79
Int_Acro-Meso	Interface between acrotelm and mesotelm	24	1.99	27.28	3.46	23.61
Meso	Mesotelm samples	44	1.45	20.62	3.71	21.57
Int_Meso-Cato	Interface between mesotelm and catotelm	56	1.84	38.23	4.35	25.18
Cato	Catotelm samples	96	1.23	37.93	4.95	24.91

## Data Availability

The original contributions presented in the study are included in the article, further inquiries can be directed to the corresponding authors.

## References

[1] Thevenot M., Dignac M.-F., Rumpel C. (2010). Fate of Lignins in Soils: A Review. Soil Biol. Biochem..

[2] Delarue F., Laggoun-Défarge F., Disnar J.R., Lottier N., Gogo S. (2011). Organic Matter Sources and Decay Assessment in a Sphagnum-Dominated Peatland (Le Forbonnet, Jura Mountains, France): Impact of Moisture Conditions. Biogeochemistry.

[3] Kolattukudy P.E., Babel W., Steinbüchel A. (2001). Polyesters in higher plants. Biopolyesters.

[4] Grasset L., Vlčková Z., Kučerík J., Amblès A. (2010). Characterization of Lignin Monomers in Low Rank Coal Humic Acids Using the Derivatization/Reductive Cleavage Method. Org. Geochem..

[5] Fabbri D., Chiavari G. (2001). Analytical Pyrolysis of Carbohydrates in the Presence of Hexamethyldisilazane. Anal. Chim. Acta.

[6] Fabbri D., Chiavari G., Prati S., Vassura I., Vangelista M. (2002). Gas Chromatography/Mass Spectrometric Characterisation of Pyrolysis/Silylation Products of Glucose and Cellulose. Rapid Commun. Mass Spectrom..

[7] Nieweś D., Biegun M., Huculak-Mączka M., Marecka K., Kaniewski M., Zieliński J., Hoffmann J. (2023). Extraction of Humic Acid from Peat and Lignite and the Thermal Behavior of Their Mixtures with Ammonium Nitrate. J. Therm. Anal. Calorim..

[8] Wang M., Li Y., Zhang Y., Hu X., Li Q., Su Y., Zhao W. (2021). Exploration of the H2O2 Oxidation Process and Characteristic Evaluation of Humic Acids from Two Typical Lignites. ACS Omega.

[9] Younes K., Laduranty J., Descostes M., Grasset L. (2017). Molecular Biomarkers Study of an Ombrotrophic Peatland Impacted by an Anthropogenic Clay Deposit. Org. Geochem..

[10] Younes K., Grasset L. (2018). Comparison of Thermochemolysis and Classical Chemical Degradation and Extraction Methods for the Analysis of Carbohydrates, Lignin and Lipids in a Peat Bog. J. Anal. Appl. Pyrolysis.

[11] Biester H., Knorr K.-H., Schellekens J., Basler A., Hermanns Y.-M. (2014). Comparison of Different Methods to Determine the Degree of Peat Decomposition in Peat Bogs. Biogeosciences.

[12] Schellekens J., Bindler R., Martínez-Cortizas A., McClymont E.L., Abbott G.D., Biester H., Pontevedra-Pombal X., Buurman P. (2015). Preferential Degradation of Polyphenols from Sphagnum–4-Isopropenylphenol as a Proxy for Past Hydrological Conditions in Sphagnum-Dominated Peat. Geochim. Cosmochim. Acta.

[13] Estournel-Pelardy C., Delarue F., Grasset L., Laggoun-Défarge F., Amblès A. (2011). Tetramethylammonium Hydroxide Thermochemolysis for the Analysis of Cellulose and Free Carbohydrates in a Peat Bog. J. Anal. Appl. Pyrolysis.

[14] Filley T.R., Minard R.D., Hatcher P.G. (1999). Tetramethylammonium Hydroxide (TMAH) Thermochemolysis: Proposed Mechanisms Based upon the Application of 13C-Labeled TMAH to a Synthetic Model Lignin Dimer. Org. Geochem..

[15] Del Río J.C., Hatcher P.G. (1998). Analysis of Aliphatic Biopolymers Using Thermochemolysis with Tetramethylammonium Hydroxide (TMAH) and Gas Chromatography–Mass Spectrometry. Org. Geochem..

[16] Challinor J.M. (1991). The Scope of Pyrolysis Methylation Reactions. J. Anal. Appl. Pyrolysis.

[17] Shadkami F., Helleur R. (2010). Recent Applications in Analytical Thermochemolysis. J. Anal. Appl. Pyrolysis.

[18] Guignard C., Lemée L., Amblès A. (2005). Lipid Constituents of Peat Humic Acids and Humin. Distinction from Directly Extractable Bitumen Components Using TMAH and TEAAc Thermochemolysis. Org. Geochem..

[19] Válková D., Grasset L., Amblès A. (2009). Molecular Compounds Generated by Ruthenium Tetroxide Oxidation and Preparative off Line Thermochemolysis of Lignite Humic Acids from South Moravia: Implications for Molecular Structure. Fuel.

[20] Chiavari G., Fabbri D., Prati S. (2001). In-Situ Pyrolysis and Silylation for Analysis of Lipid Materials Used in Paint Layers. Chromatographia.

[21] Scalarone D., Chiantore O., Riedo C. (2008). Gas Chromatographic/Mass Spectrometric Analysis of on-Line Pyrolysis–Silylation Products of Monosaccharides. J. Anal. Appl. Pyrolysis.

[22] Kuroda K.-I. (2000). Pyrolysis-Trimethylsilylation Analysis of Lignin: Preferential Formation of Cinnamyl Alcohol Derivatives. J. Anal. Appl. Pyrolysis.

[23] Filley T.R., Nierop K.G., Wang Y. (2006). The Contribution of Polyhydroxyl Aromatic Compounds to Tetramethylammonium Hydroxide Lignin-Based Proxies. Org. Geochem..

[24] Hedges J.I., Mann D.C. (1979). The Characterization of Plant Tissues by Their Lignin Oxidation Products. Geochim. Cosmochim. Acta.

[25] Otto A., Simpson M.J. (2006). Evaluation of CuO Oxidation Parameters for Determining the Source and Stage of Lignin Degradation in Soil. Biogeochemistry.

[26] Tareq S.M., Tanaka N., Ohta K. (2004). Biomarker Signature in Tropical Wetland: Lignin Phenol Vegetation Index (LPVI) and Its Implications for Reconstructing the Paleoenvironment. Sci. Total Environ..

[27] Opsahl S., Benner R. (1995). Early Diagenesis of Vascular Plant Tissues: Lignin and Cutin Decomposition and Biogeochemical Implications. Geochim. Cosmochim. Acta.

[28] Ertel J.R., Hedges J.I. (1985). Sources of Sedimentary Humic Substances: Vascular Plant Debris. Geochim. Cosmochim. Acta.

[29] Hedges J.I., Clark W.A., Come G.L. (1988). Organic Matter Sources to the Water Column and Surficial Sediments of a Marine Bay: Organic Matter Sources. Limnol. Oceanogr..

[30] Hu J., Wang Q., Zhang Y., Meng Z., Zhang J., Fan J. (2023). Numerical and Experimental Study on the Process of Filling Water in Pressurized Water Pipeline. Water.

[31] da Silva Oliveira D.M., Schellekens J., Cerri C.E.P. (2016). Molecular Characterization of Soil Organic Matter from Native Vegetation–Pasture–Sugarcane Transitions in Brazil. Sci. Total Environ..

[32] Kaal J., Serrano O., Nierop K.G., Schellekens J., Cortizas A.M., Mateo M.-Á. (2016). Molecular Composition of Plant Parts and Sediment Organic Matter in a Mediterranean Seagrass (*Posidonia oceanica*) Mat. Aquat. Bot..

[33] Schellekens J., Buurman P., Pontevedra-Pombal X. (2009). Selecting Parameters for the Environmental Interpretation of Peat Molecular Chemistry—A Pyrolysis-GC/MS Study. Org. Geochem..

[34] Schellekens J., Horak-Terra I., Buurman P., Silva A.C., Vidal-Torrado P. (2014). Holocene Vegetation and Fire Dynamics in Central-Eastern Brazil: Molecular Records from the Pau de Fruta Peatland. Org. Geochem..

[35] Joliffe I., Morgan B. (1992). Principal Component Analysis and Exploratory Factor Analysis. Stat. Methods Med. Res..

[36] Clymo R.S., Bryant C.L. (2008). Diffusion and Mass Flow of Dissolved Carbon Dioxide, Methane, and Dissolved Organic Carbon in a 7-m Deep Raised Peat Bog. Geochim. Cosmochim. Acta.

[37] Boekhout F., Gérard M., Kanzari A., Michel A., Déjeant A., Galoisy L., Calas G., Descostes M. (2015). Uranium Migration and Retention during Weathering of a Granitic Waste Rock Pile. Appl. Geochem..

[38] Cameron C.C., Esterle J.S., Palmer C.A. (1989). The Geology, Botany and Chemistry of Selected Peat-Forming Environments from Temperate and Tropical Latitudes. Int. J. Coal Geol..

[39] Grasset L., Amblès A. (1998). Structural Study of Soil Humic Acids and Humin Using a New Preparative Thermochemolysis Technique. J. Anal. Appl. Pyrolysis.

[40] Younes K., Mouhtady O., Chaouk H., Obeid E., Roufayel R., Moghrabi A., Murshid N. (2021). The Application of Principal Component Analysis (PCA) for the Optimization of the Conditions of Fabrication of Electrospun Nanofibrous Membrane for Desalination and Ion Removal. Membranes.

[41] Younes K., Moghrabi A., Moghnie S., Mouhtady O., Murshid N., Grasset L. (2022). Assessment of the Efficiency of Chemical and Thermochemical Depolymerization Methods for Lignin Valorization: Principal Component Analysis (PCA) Approach. Polymers.

